# Supplementary material for: Strain-tunable Berry curvature in quasi-two-dimensional chromium telluride
Source: Nat Commun. 2023 Jun 3;14:3222. doi: 10.1038/s41467-023-38995-4 (PMC10239464; doi:10.1038/s41467-023-38995-4)
Supplement: Supplementary file 1 — Supplementary Information [file 41467_2023_38995_MOESM1_ESM.pdf]

Supplementary Information for
Strain-tunable Berry curvature in quasi-two-dimensional chromium telluride

Hang Chi,[1,2] Yunbo Ou,[1] Tim B. Eldred,[3] Wenpei Gao,[3] Sohee Kwon,[4] Joseph Murray,[5] Michael Dreyer,[5] Robert E. Butera,[6] Alexandre C. Foucher,[7] Haile Ambaye,[8] Jong Keum,[8,9] Alice T. Greenberg,[2] Yuhang Liu,[4] Mahesh R. Neupane,[2,4] George J. de Coster,[2] Owen A. Vail,[2] Patrick J. Taylor,[2] Patrick A. Folkes,[2] Charles Rong,[2] Gen Yin,[10] Roger K. Lake,[4] Frances M. Ross,[7] Valeria Lauter,[8] Don Heiman,[1,11] and Jagadeesh S. Moodera[1,12]

[1]*Francis Bitter Magnet Laboratory, Plasma Science and Fusion Center, Massachusetts Institute of Technology, Cambridge, Massachusetts 02139, USA*
[2]*U.S. Army DEVCOM Army Research Laboratory, Adelphi, Maryland 20783, USA*
[3]*Department of Materials Science and Engineering, North Carolina State University, Raleigh, North Carolina 27695, USA*
[4]*Department of Electrical and Computer Engineering, University of California, Riverside, California 92521, USA*
[5]*Department of Physics, University of Maryland, College Park, Maryland 20742, USA*
[6]*Laboratory for Physical Sciences, College Park, Maryland 20740, USA*
[7]*Department of Materials Science and Engineering, Massachusetts Institute of Technology, Cambridge, Massachusetts 02139, USA*
[8]*Neutron Scattering Division, Neutron Sciences Directorate, Oak Ridge National Laboratory, Oak Ridge, Tennessee 37831, USA*
[9]*Center for Nanophase Materials Sciences, Physical Science Directorate, Oak Ridge National Laboratory, Oak Ridge, Tennessee 37831, USA*
[10]*Department of Physics, Georgetown University, Washington, District of Columbia 20057, USA*
[11]*Department of Physics, Northeastern University, Boston, Massachusetts 02115, USA*
[12]*Department of Physics, Massachusetts Institute of Technology, Cambridge, Massachusetts 02139, USA*

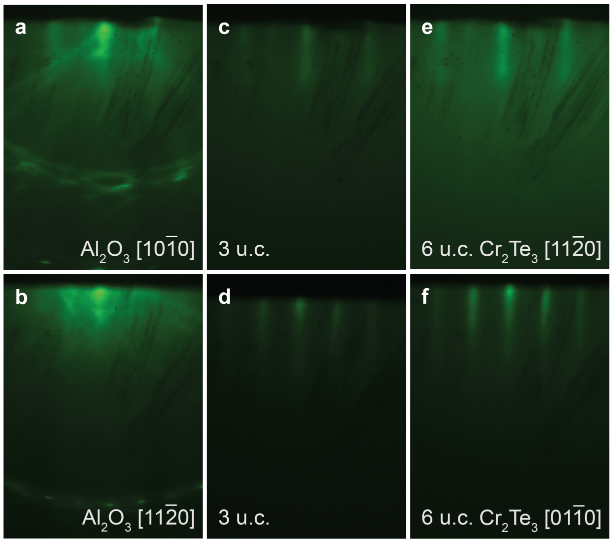

Supplementary Figure 1 | Reflection high-energy electron diffraction of Cr_2Te_3. Typical *in situ* RHEED patterns from the surface of heat-treated Al_2O_3(0001) substrates (**a**, **b**) and as-grown *c*-oriented Cr_2Te_3 (**c**-**f**) with thickness of 3 (**c**, **d**) and 6 (**e**, **f**) unit cell (u.c.). The incident electron beam is along the [$10\bar{1}0$] (**a**, **c**, **e**) and [$11\bar{2}0$] (**b**, **d**, **f**) crystalline orientations of Al_2O_3, respectively. The clear Kikuchi lines in **a** and **b** attest to an atomically flat surface ready for the fabrication of high-quality epitaxial films. The corresponding RHEED patterns from the *c*-oriented Cr_2Te_3 (001) surface reveal sharp and streaky diffraction during the film deposition process, indicating the formation of a highly ordered and smooth surface as well as a 2D growth mode. Upon in-plane rotation, the same RHEED patterns reemerge every 60°, suggesting a six-fold crystalline symmetry within the basal plane of the as-grown films.

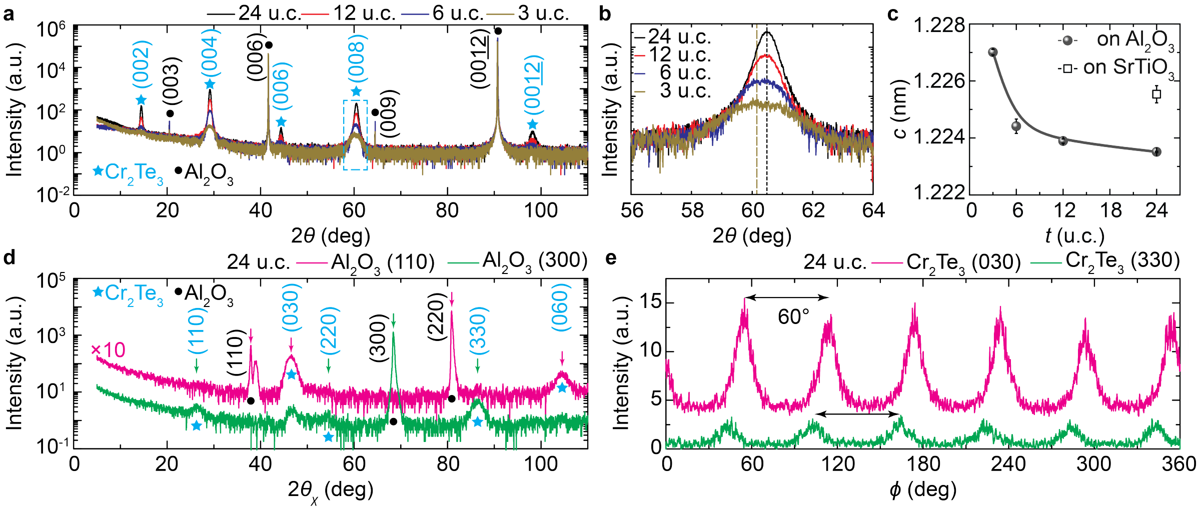

Supplementary Figure 2 | X-ray diffraction of Cr_2Te_3. **a**, Out-of-plane $2\theta/\omega$ XRD patterns of c-oriented Cr_2Te_3 on Al_2O_3(0001) with thickness $t = 3 - 24$ u.c.. **b**, Enlarged view of the (008) peaks showing gradual shift towards lower 2θ upon decreasing t. **c**, The t-dependence of the c lattice parameter indicating enhanced in-plane compressive strain at reduced t. **d**, In-plane $2\theta_\chi/\phi$ scans for $t = 24$ u.c. Cr_2Te_3 thin film, aligned with the Al_2O_3 (110) and (300) orientations. **e**, X-ray ϕ scans with $2\theta_\chi$ angle fixed at Cr_2Te_3 (030) and (330), respectively, corroborating the in-plane six-fold rotational symmetry. The lattice parameters are measured to be $a = b = 0.675$ ($\pm$ 0.002) nm and $c = 1.223$ ($\pm$ 0.004) nm, respectively, for $t = 24$ u.c..

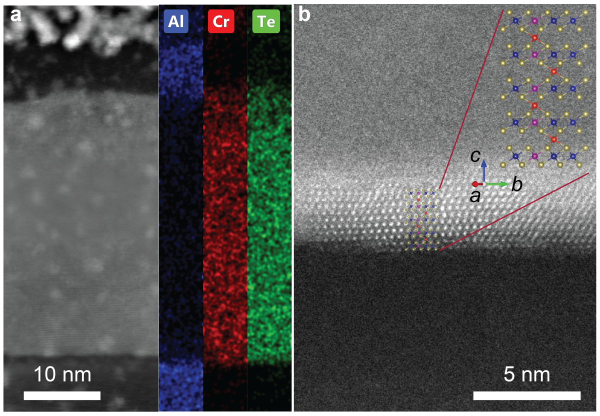

Supplementary Figure 3 | Scanning transmission electron microscopy of Cr_2Te_3. **a**, Cross sectional STEM imaging of c-oriented Cr_2Te_3 on Al_2O_3(0001) with thickness $t = 24$ u.c.. The energy dispersive X-ray spectroscopy (EDS) profile reveals a uniform elemental distribution. **b**, HAADF STEM image of a $t = 3$ u.c. sample illustrating the film quality.

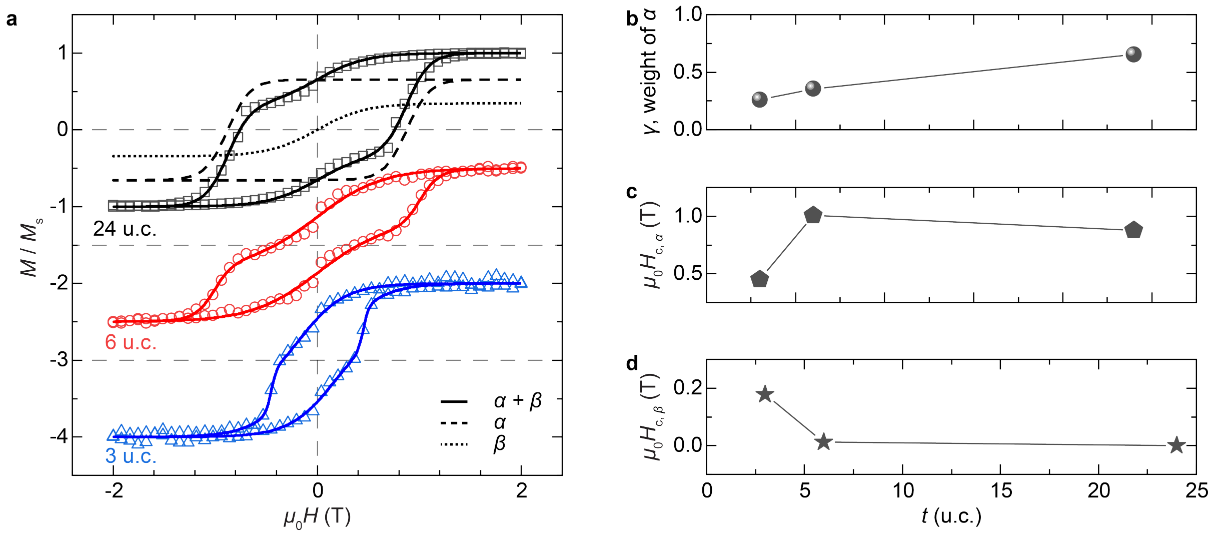

Supplementary Figure 4 | Interface-driven two component magnetic switching in Cr_2Te_3. **a**, Field dependence of normalized magnetization M/M_s at 2 K, under the out-of-plane (OOP) configuration for t = 24, 6 and 3 u.c. (black, red and blue open symbols, vertically shifted for clarity), respectively. The two-component numerical model (solid line) depicts well the contributions from α (dashed line) and β (dotted line) layers with different magnetic anisotropies modulated by interfacial strain, via $M/M_s \sim \gamma \tanh[\alpha(H + H_{c,\alpha})] + (1 - \gamma) \tanh[\beta(H + H_{c,\beta})]$. **b-d**, Thickness dependence of γ, the relative weight of the magnetically harder top α layer, as well as the coercive fields $H_{c,\alpha}$ (**c**) and $H_{c,\beta}$ (**d**), reveals the more dominant role of the more strongly strained bottom β layer at reduced thickness t.

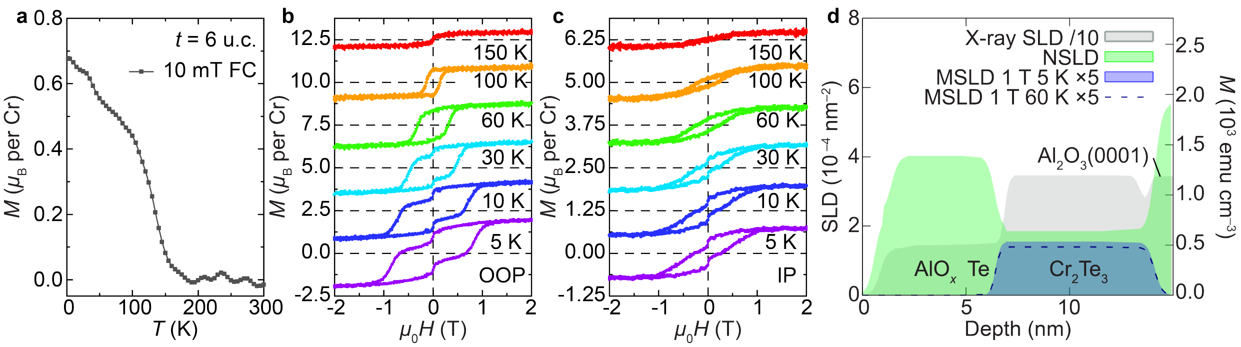

Supplementary Figure 5 | Magnetization of 6 u.c. Cr_2Te_3. **a**, Temperature dependence of the magnetization M of 6 u.c. Cr_2Te_3 film under the field-cool (FC) condition with an out-of-plane (OOP) magnetic field μ_0H = 10 mT. **b-c**, Field dependence of M under OOP (**b**) and in-plane (IP, **c**) configurations, respectively, at selected T. Curves are vertically shifted for clarity. The preference of perpendicular magnetic anisotropy (PMA) is evident in the OOP $M(H)$ scans. **d**, Depth profiles of polarized neutron reflectometry (PNR) nuclear (NSLD), magnetic (MSLD, with IP μ_0H = 1 T at 5 K and 60 K, respectively) and X-ray scattering length densities (SLD) of 6 u.c. Cr_2Te_3 on Al_2O_3(0001) substrate with Te/AlO_x capping.

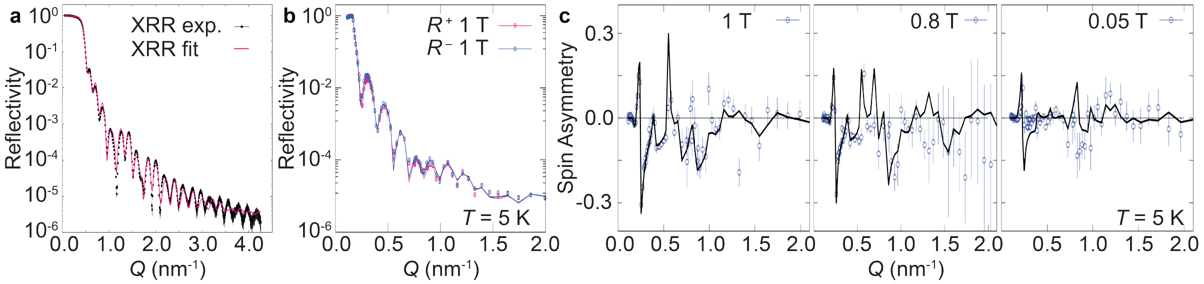

Supplementary Figure 6 | X-ray and polarized neutron reflectivity of 24 u.c. Cr_2Te_3 on Al_2O_3(0001). **a**, Measured (points) and fitted (lines) X-ray reflectivity. **b**, Polarized neutron reflectivity at T = 5 K and μ_0H = 1 T. **c**, The PNR spin asymmetry ratio SA $= (R^+ - R^-)/(R^+ + R^-)$ for μ_0H = 1 T, 0.8 T and 0.05 T, respectively.

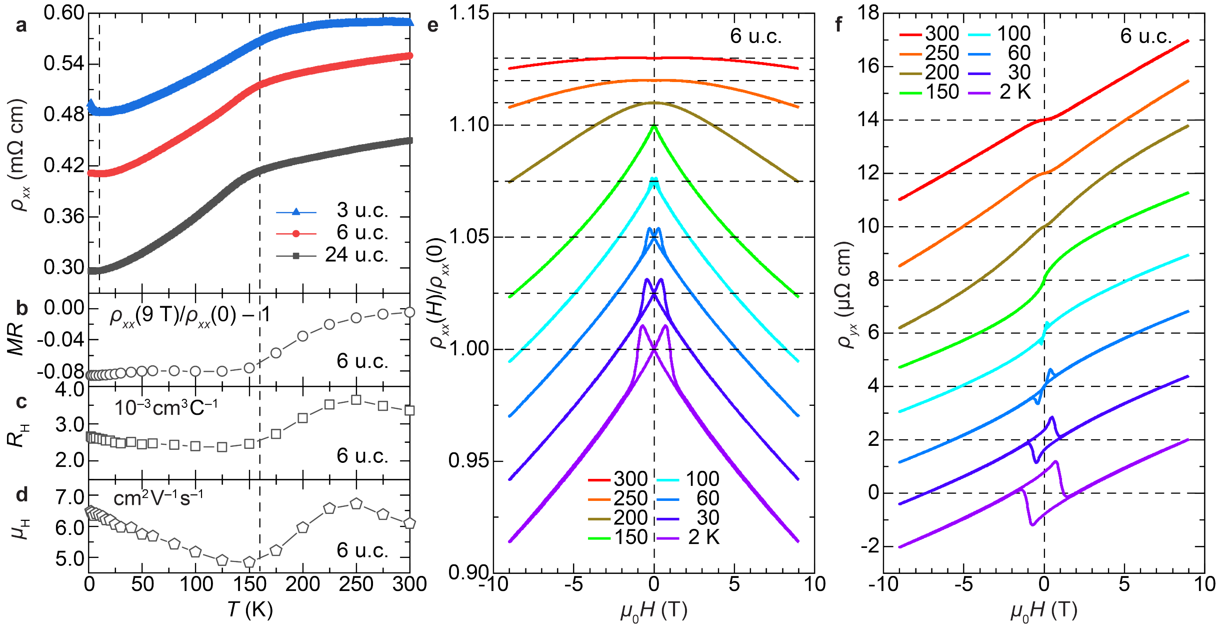

Supplementary Figure 7 | Transport properties of Cr_2Te_3 thin films. **a**, Temperature dependence of the longitudinal electrical resistivity $\rho_{xx}(T)$ of Cr_2Te_3 with t = 3 – 24 u.c.. **b-d**, The key transport parameters for t = 6 u.c., namely, the magnetoresistance $[MR \equiv \rho_{xx}(H)/\rho_{xx}(0) - 1]$ at $\mu_0 H$ = 9 T (**b**), the Hall coefficient R_{H} (**c**) and the Hall mobility μ_{H} (**d**) derived from the linear ordinary Hall effect at 8 – 9 T. **e-f**, Magnetic field dependence of $\rho_{xx}(H)$ (**e**) and the Hall resistivity $\rho_{yx}(H)$ (**f**) at selected temperatures for t = 6 u.c.. Curves in **e-f** are shifted vertically for clarity.

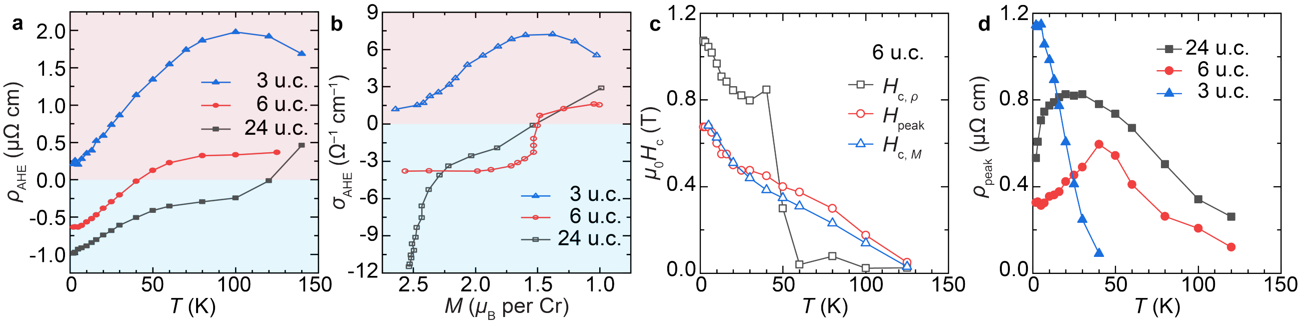

Supplementary Figure 8 | The characteristics of the unconventional Hall response of Cr_2Te_3. **a**, Temperature dependence of ρ_{AHE} for t = 24 u.c. (black), 6 u.c. (red) and 3 u.c. (blue). **b**, The corresponding anomalous Hall conductivity σ_{AHE} as a function of the magnetization M. **c**, Temperature dependence of the field H_{peak} at which the hump-shaped peak occurs (red) for t = 6 u.c., along with the coercive fields $H_{c,\rho}$ (black) and $H_{c,M}$ (blue) determined from transport and magnetization experiments, respectively. **d**, Temperature dependence of the magnitude of the hump-shaped Hall feature ρ_{peak}.

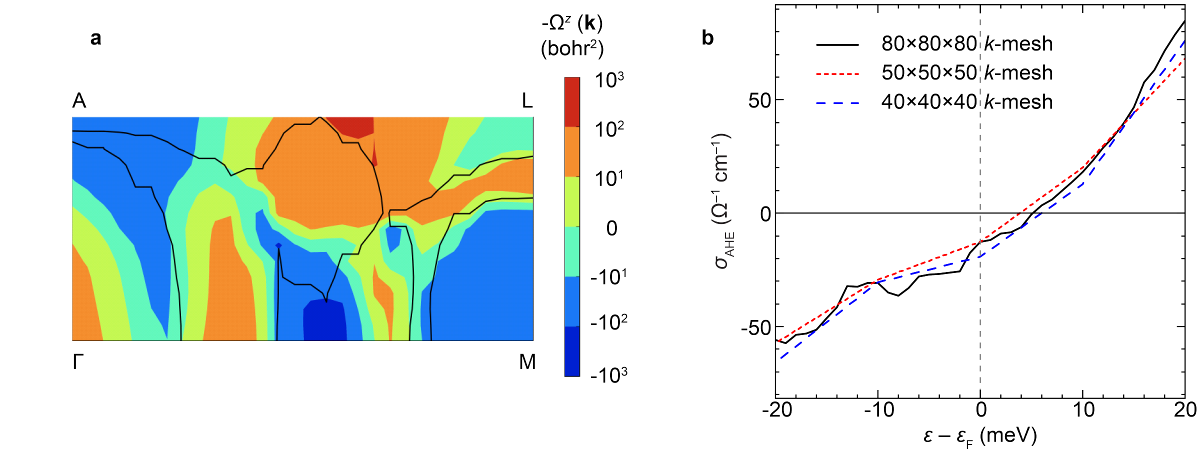

Supplementary Figure 9 | Berry curvature and anomalous Hall conductivity in Cr_2Te_3. **a**, Berry curvature contour plot of $-\Omega^z(\mathbf{k})$ with Fermi surface in the Γ-M-L-A k-plane. **b**, Convergence test of k-mesh for energy dependent $\sigma_{\rm AHE}$ near the Fermi level $\varepsilon_{\rm F}$.

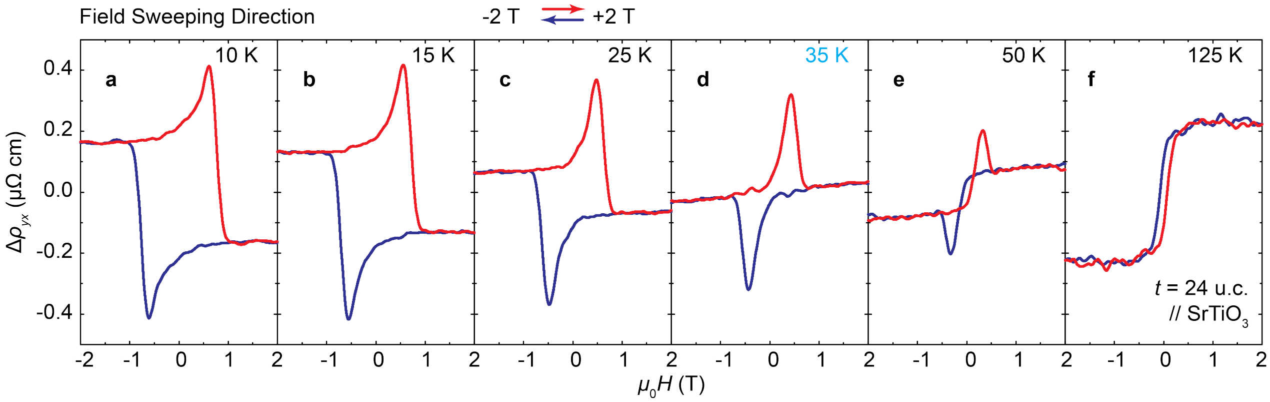

Supplementary Figure 10 | Hall response of 24 u.c. Cr_2Te_3 grown on $SrTiO_3(111)$. The Hall traces after removing the linear background at 10 K (**a**), 15 K (**b**), 25 K (**c**), 35 K (**d**), 50 K (**e**) and 125 K (**f**), respectively. Despite the choice of different substrate and hence distinct interface conditions, the temperature dependent anomalous Hall effect sign reversal and the unconventional hump-shaped Hall peaks are also present, attesting to the universality of the observed phenomena.
